# Neuroprotective effects of a *Coeloglossum viride* var. Bracteatum extract *in vitro* and *in vivo*

**DOI:** 10.1038/s41598-017-08957-0

**Published:** 2017-08-23

**Authors:** Rui-Yuan Pan, Jun Ma, Huan-Tong Wu, Qing-Shan Liu, Xiao-Yan Qin, Yong Cheng

**Affiliations:** 1 Center on Translational Neuroscience, College of Life & Environmental Science, Minzu University of China, Beijing, 100081 China; 2 State Key Laboratory of Brain and Cognitive Sciences, Institute of Biophysics, Chinese Academy of Sciences, Beijing, 100101 China; 3 College of Life Sciences, University of Chinese Academy of Sciences, Beijing, 100049 China

## Abstract

The excessive release and accumulation of glutamate in the brain is known to be associated with excitotoxicity. CE, an extract derived from the plant *Coeloglossum viride* var. Bracteatum, exerted neuroprotective effects against amyloid toxicity and oxidative stress in cortical neurons. The aims of this study are to examine whether CE also attenuates glutamate neurotoxicity in rat primary cultured cortical neurons and to determine the effect of CE *in vivo*. According to the results of MTT, LDH release, and TUNEL assays, the CE treatment significantly reduced glutamate-induced neurotoxicity in a dose-dependent manner. Moreover, the protective effects of CE were blocked by an Akt inhibitor, LY294002, suggesting that the PI3K/Akt signalling pathway is involved in the neuroprotective effects of CE. In addition, CE might regulate the PKC-GluA2 axis to prevent neuronal apoptosis. CE also protected against dopaminergic neuronal loss in a mouse model of MPTP-induced PD. Based on our results, CE exerted neuroprotective effects both *in vitro* and *in vivo*, thus providing a potential therapeutic target for the treatment or prevention of neurodegeneration.

## Introduction

Glutamate is a major endogenous excitatory neurotransmitter in the mammalian central nervous system, particularly the hippocampus and cortex, and therefore plays an essential role in many neurophysiological processes, such as neuronal development, synaptogenesis, synaptic plasticity, learning and memory^1^. However, the excessive release and accumulation of glutamate will lead to neuronal dysfunction and neuronal loss by apoptosis and necrosis, which is now referred to as “glutamate neurotoxicity” and is generally believed to be involved in the development of certain neurodegenerative diseases, including Parkinson’s disease (PD), Alzheimer’s disease (AD) and Huntington’s disease (HD)^2, 3^. Therefore, treatments that protect against glutamate-induced neurotoxicity are considered potential therapeutic strategies for certain neurodegenerative diseases.

CE is an extract derived from an Orchidaceae family plant called *Coeloglossum viride* var. Bracteatum. CE is a Chinese traditional medicine that is widely used in northwest regions of China, such as the Tibet, Gansu, Qinghai, Shanxi and Inner Mongolia provinces. In 2004, CE was identified as a mixture of 4 compounds (dactylorhin A, dactylorhin B, loroglossin and militarine)^4^. CE treatment was reported to significantly increase vital energy and body fluid production; it was also reported to exert beneficial effects on memory and tranquilization effects^5^. The traditional Chinese medicine CE rescues scopolamine-induced learning and memory dysfunction in rodents^6^. Moreover, based on the results from our previous studies, CE exerts neuroprotective effects against amyloid toxicity and oxidative stress in cortical neurons^7, 8^, indicating that CE may be a promising medicine for the treatment of neurodegenerative diseases.

We aimed to examine whether CE also has neuroprotective effects against glutamate-induced excitotoxicity in this study to expand the potential applications of CE. In addition, a mouse model of MPTP-induced PD was also used to investigate the effect of CE *in vivo*. Glutamate-induced death of rat primary cortical neurons was completely prevented by a CE treatment. The neuroprotective effect of CE may be mediated by the activation of the Akt signalling pathway that regulates the expression levels of an anti-apoptotic protein, Bcl-2, and an apoptotic protein, caspase-3. In addition, CE increased p-PKC levels, which then phosphorylated its downstream target GluA2. Moreover, CE protected against dopaminergic neuronal loss induced by the MPTP treatment *in vivo*. Thus, based on our results, CE is a promising target neurodegenerative disease therapy.

## Materials and Methods

### Primary cortical neuron culture

Primary cortical neuron cultures were prepared using a previously described method, and the neuronal purity is approximately 90%^9^. Briefly, cortical tissues were dissected from whole brains of new-born Sprague-Dawley (SD) rats, mechanically dissociated, and then digested with 0.25% trypsin (Invitrogen) for 20 minutes at 37 °C. The digestion was terminated by the addition of 10% foetal bovine serum (FBS) in DMEM. After filtration through nylon meshes, the cells were centrifuged and resuspended in DMEM supplemented with 10% FBS, 2 g/L HEPES, penicillin G (100 U/ml), and 100 μg/ml streptomycin (Invitrogen). Cells were plated on poly-L-lysine-coated plates or coverslips and cultured in a 37 °C cell incubator with 5% CO_2_, 95% O_2_. Cells were treated with 10 µM cytosine arabinoside (Sigma) for 3 days after plating to inhibit glial cell growth. Cells were cultured for 7–8 days before use. All methods were performed in accordance with the guidelines approved by the Animal Care and Use Committee of Minzu University of China.

### Drug treatments

After 7 days in culture, different concentrations of CE (a gift from Dr. Li Tang, Department of Pharmacology, Minzu University of China) were freshly added to the culture medium of primary cortical neurons for 24 hours; the neurons were then treated with glutamate for 24 hours. In some experiments, cells were pretreated with an Akt inhibitor (LY29004, final concentration is 10 μM) or PKC inhibitor (Bisindolylmaleimide I, BIM, final concentration is 0.5 μM) or Caspase-3 inhibitor (DEVD, final concentration is 0.4 μM)for 30 min prior to treatment with CE or glutamate.

For the mouse model of MPTP-induced PD, adult C57BL/6 J mice (8–10 weeks) were administered 20 mg/kg MPTP (four intraperitoneal injections) as previously described^10, 11^. Mice were intragastrically administered 10 mg/kg CE or saline once a day until sacrifice to explore the neuroprotective effect of CE. After 7 days of MPTP treatments, mice were sacrificed and the striatum and ventral midbrain were dissected and processed for western blot analysis. In some cases, mice were perfused with 4% paraformaldehyde (PFA), and coronal sections were prepared for immunohistochemistry assays.

### Measurements of cell death: MTT, LDH release, and TUNEL assays

The viability of cells exposed to various treatments was estimated by measuring their ability to reduce the dye methyl thiazolyl tetrazolium (MTT, Sigma) to blue formazan crystals. Cells that had been cultured in 96-well plates for 7 days were gently washed with 0.01 M PBS. After washing, 90 μl of medium with 10 μl of a 5 mg/ml MTT solution were added to each well and the plate was incubated at 37 °C for 1–2 hours. Then, the crystals were dissolved in DMSO and the absorption was measured at 570 nm using a microplate spectrophotometer (Bio-Rad); the results represented the relative cell viability.

The cytotoxicity of cells exposed to various treatments was evaluated by measuring lactate dehydrogenase (LDH) release using a CytoTox96® Non-Radioactive Cytotoxicity Assay kit (Promega), according to the manufacturer’s instructions.

Cell death was examined after treatments by fixing cells with fresh 4% PFA and 4% sucrose in PBS for 20 minutes at room temperature, followed by permeabilization in 0.1% Triton X-100 and 0.1% sodium citrate in PBS for 2 minutes on ice. Terminal deoxynucleotidyl transferase-mediated dUTP nick-end labelling (TUNEL) staining was performed using the *in situ* cell death detection kit I (Roche), according to the manufacturer’s instructions. Coverslips were then washed once with distilled water for 5 minutes and mounted on glass slides to be observed under a fluorescence microscope. The percentage of cell death was determined by calculating the ratio of the number of TUNEL-positive cells to a total of 100 cells in one count. The average of 5 counts was calculated as the percentage of neuronal cell death in response to a certain treatment.

### Western blots

Western blotting was performed as previously reported^9^. Briefly, total proteins were extracted from the cultured neurons using cell lysis buffer containing 1% phenylmethylsulfonyl fluoride (PMSF). The protein concentration was measured using BCA assays and adjusted to the same final concentration. The lysate samples were separated by SDS-PAGE and then transferred to a polyvinylidene fluoride (PVDF, Millipore) membrane. After blocking with 5% non-fat milk in Tris-buffered saline for 1 hour, the membrane was then incubated with primary antibodies overnight at 4 °C. After washing, the membrane was incubated with horseradish peroxidase (HRP)-conjugated secondary antibodies (Jackson ImmunoResearch Laboratories). The primary antibodies used in this study include: β-actin (Santa Cruz Biotechnology), cleaved-Csp3 (Cell Signaling Technology), Bcl-2 (Santa Cruz Biotechnology), Akt (Cell Signaling Technology), p-Akt (S473) (Cell Signaling Technology), p-PKCα (S657) (Santa Cruz Biotechnology), PKCα (Santa Cruz Biotechnology), p-GluA1 (S845) (Abcam), GluA2 (Epitomics), p-GluA2 (S880) (Abcam) and TH (Pel Freez Biologicals). Finally, the lane intensity was scanned and quantified using ImageJ software.

### Immunofluorescence and immunohistochemistry

Cortical neurons cultured on coverslips in 24-well plates were used for immunofluorescence staining after exposure to different treatments. Briefly, cortical neurons were fixed with 4% paraformaldehyde (PFA) at room temperature for 15 min. Then, the fixed primary cortical neurons were incubated with blocking buffer (PBS containing 5% serum and 0.3% Triton-X-100) at room temperature for 1 h, followed by an incubation with the MAP2 primary antibody (1:500, Sigma) overnight at 4 °C. After 3 washes with PBS, the coverslips with neurons were incubated with an Alexa Fluor® 546-conjugated secondary antibody (Invitrogen) at room temperature for 1.5 h. The coverslips were then washed with PBS 3 times and the nuclei were stained with Hoechst 33258 (Sigma) at room temperature for 15 min. Images were acquired with a Leica confocal microscope.

Mouse brains were post-fixed with 4% PFA, cryoprotected in 30% sucrose, and processed for immunohistochemistry. Briefly, the brains were sectioned at 40 μm using a freezing microtome (Leica). Sections were incubated with 3% H_2_O_2_ for 15 min to inhibit endogenous peroxidases and sections were washed with PBS 3 times. Then, sections were incubated with blocking buffer (PBS containing 5% serum and 0.3% Triton-X-100) for 1 h, followed by the primary anti-tyrosine hydroxylase (TH) antibody (1:1000, Pel Freez Biologicals) overnight at 4 °C. Subsequently, the signal was detected with DAB using Vectastain ABC kits (Vector Laboratories), according to the manufacturer’s instructions. After mounting on a glass slide, sections were dehydrated with increasing concentrations of ethanol (50%, 75%, 85%, 95% and 100%), cleared with xylene, and cover-slipped with resinene. Finally, sections were imaged using an upright Leica microscope.

### Statistical evaluation

Results are presented as means ± S.E.M. Statistical significance (*p < 0.05, **p < 0.01 or ***p < 0.001) among groups was determined with a One-way ANOVA followed by Tukey’s multiple comparisons test using GraphPad Prism software.

## Results

### CE prevents glutamate-induced excitotoxicity in cultured primary cortical neurons

**Figure 1:**
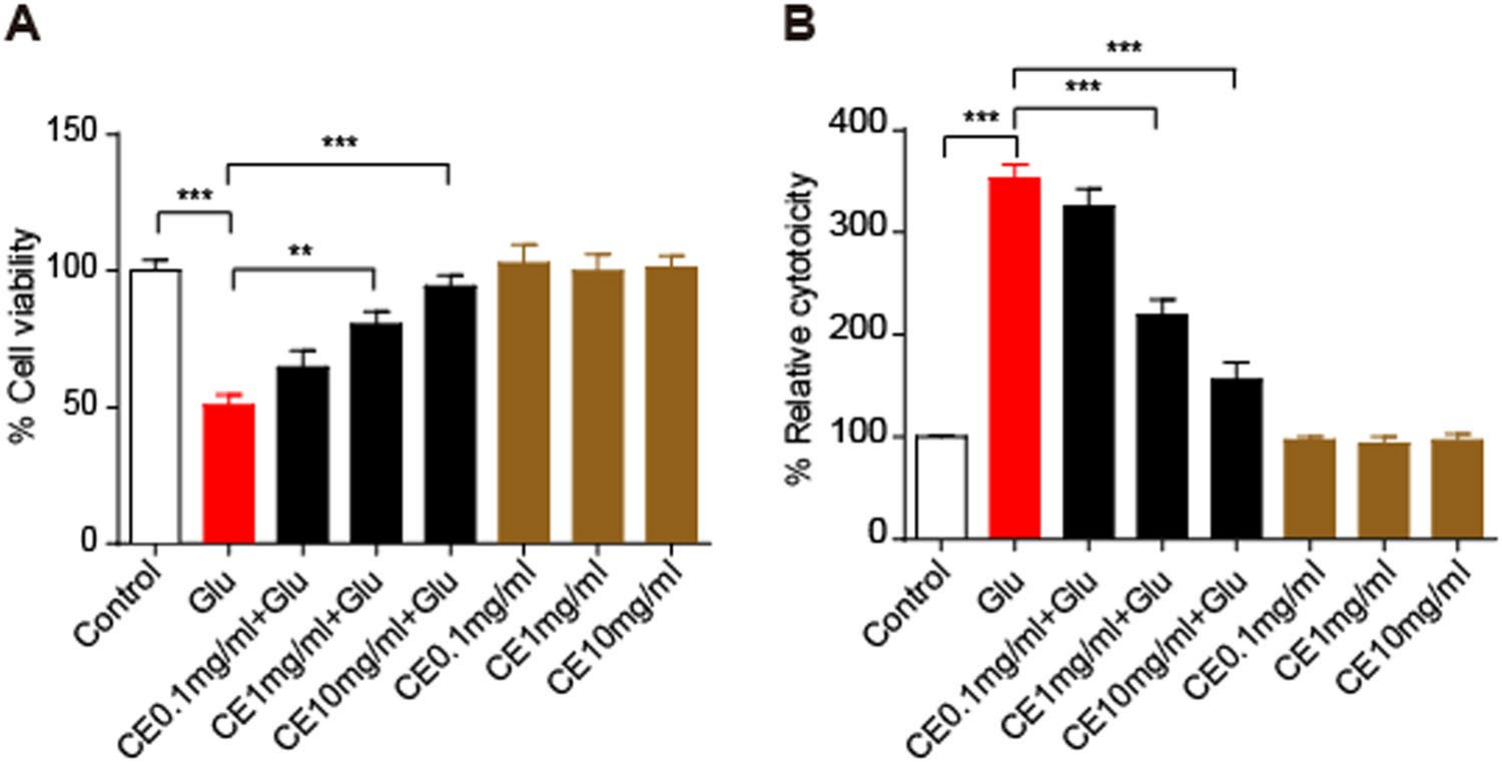
CE protects against glutamate-induced neurotoxicity. Primary cortical neurons were cultured for 7 days and then treated with 0.1, 1 and 10 mg/ml CE in the presence or absence of 100 μM glutamate for 24 hours. (**A**) Cell viability was measured using the MTT assay (n = 5). (**B**) Cytotoxicity was tested using the LDH release assay (n = 5). Glu, glutamate. Data are shown as mean ± SEM from 3 individual experiments; One-way ANOVA followed by Tukey’s multiple comparisons test. **p < 0.01, ***p < 0.001.

**Figure 2:**
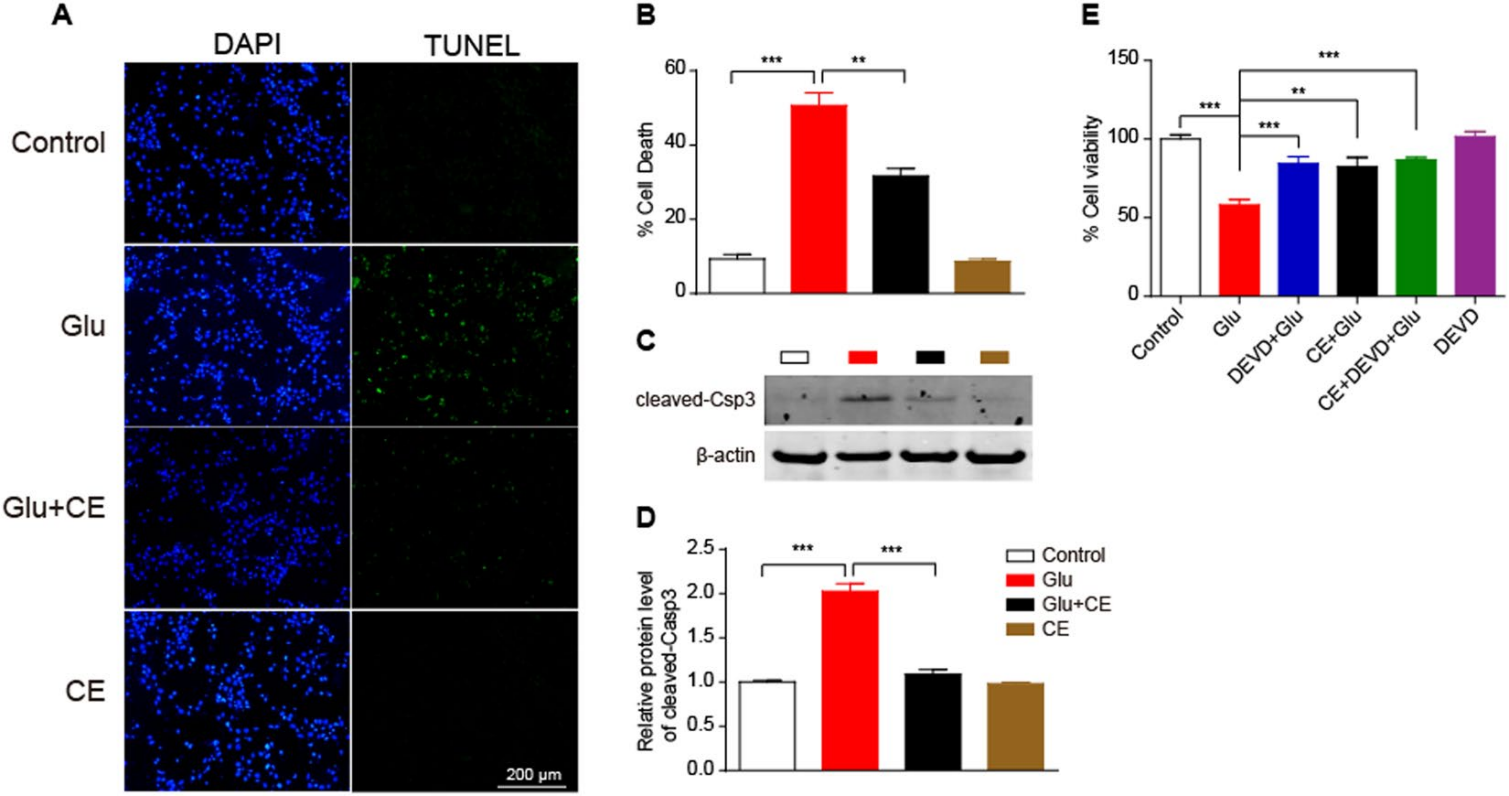
Glutamate-induced neuronal apoptosis is inhibited by CE. (**A**) Neuronal apoptosis was measured using the TUNEL assay. Drug treatments were the same as in Fig. 1, but a final CE concentration of 10 mg/ml was used due to its better protective effect. The nuclei were stained with DAPI. (**B**) Quantification of the percentage of dead cells (TUNEL-positive cells/DAPI-positive cells). At least 100 cells from each group were counted. (**C** and **D**) Cell lysates were immunoblotted with an anti-cleaved-caspase3 antibody. The protein expression levels shown in (**D**) were quantified using ImageJ software. cleaved-csp3, cleaved-caspase3. (**E**) cell viability was measured using MTT assay. Cells were pretreated with the caspase-3 inhibitor DEVD for 30 min prior to treatment with CE or glutamate. (**B** and **D**) n = 3; E, n = 6, data are shown as mean ± SEM; One-way ANOVA followed by Tukey’s multiple comparisons test. **p < 0.01, ***p < 0.001. Scale bar, 200 μm.

**Figure 3:**
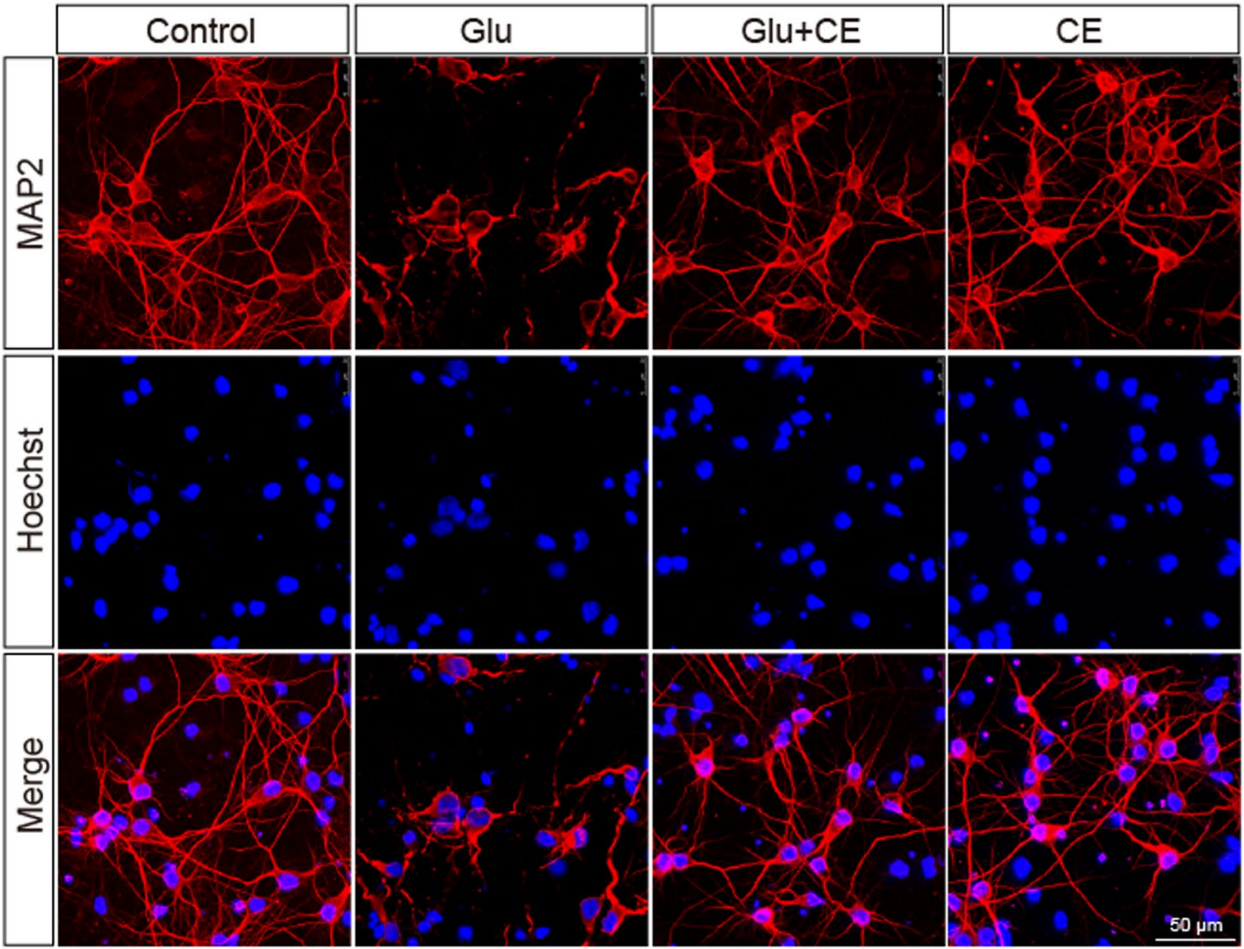
The morphological integrity of neurons is rescued by CE. Immunofluorescence staining was performed using an anti-MAP2 antibody, followed by an Alexa Fluor® 546-conjugated secondary antibody. Nuclei were co-immunostained with Hoechst 33258. Images were acquired using a Leica confocal microscope. Three independent experiments were performed; at least 20 photomicrographs from each group were analysed. Scale bar, 50 μm.

Primary cortical neuron cultures were pretreated with CE for 24 h and then exposed to glutamate (100 μM) for an additional 24 h to determine whether CE prevented glutamate-induced excitotoxicity. Cell viability was measured using the MTT assay and cytotoxicity was measured using the LDH release assay. Pretreatment with 1 mg/ml or 10 mg/ml CE dramatically rescued glutamate-induced excitotoxicity in neurons, but a lower concentration of CE (0.1 mg/ml) did not exert neuroprotective effect (Fig. 1A). However, treatment with CE alone did not significantly change the viability of the neurons (Fig. 1A). Similar results were observed in the LDH release assay (Fig. 1B). Results from the TUNEL assay further confirmed the neuroprotective effect of CE against glutamate-induced excitotoxicity. As shown in Fig. 2A and B, the number of apoptotic neurons was significantly increased after treatment with glutamate, and the 10 mg/ml CE pretreatment significantly prevented glutamate-induced cell death. These results are consistent with the changes in the levels of the apoptosis marker cleaved caspase-3. The glutamate treatment increased the cleaved caspase-3 levels in neurons, whereas the CE treatment reversed the glutamate-induced change (Fig. 2C and D). In order to determine whether CE inhibits glutamate-induced excitotoxicity through caspase-3, a caspase-3 inhibitor DEVD was used. Our results showed that inhibition of caspase-3 activity rescued the cell viability against glutamate-induced cell death (Fig. 2E), and treatment of CE did not enhance the protective effect of DEVD against glutamate-induced excitotoxicity, suggesting that CE confers neuroprotection by inhibiting caspase-3 activity. Moreover, the morphological integrity of cultured primary cortical neurons was measured by labelling the cells with an antibody against a neuronal marker, microtubule-associated protein 2 (MAP2). Again, the 10 mg/ml CE treatment significantly protected the neurons from glutamate toxicity (Fig. 3). Taken together, the above results revealed a protective effect of CE against glutamate-induced excitotoxicity in cultured rat primary cortical neurons.

### Involvement of the PI3K/Akt pathway in the neuroprotective effect of CE

**Figure 4:**
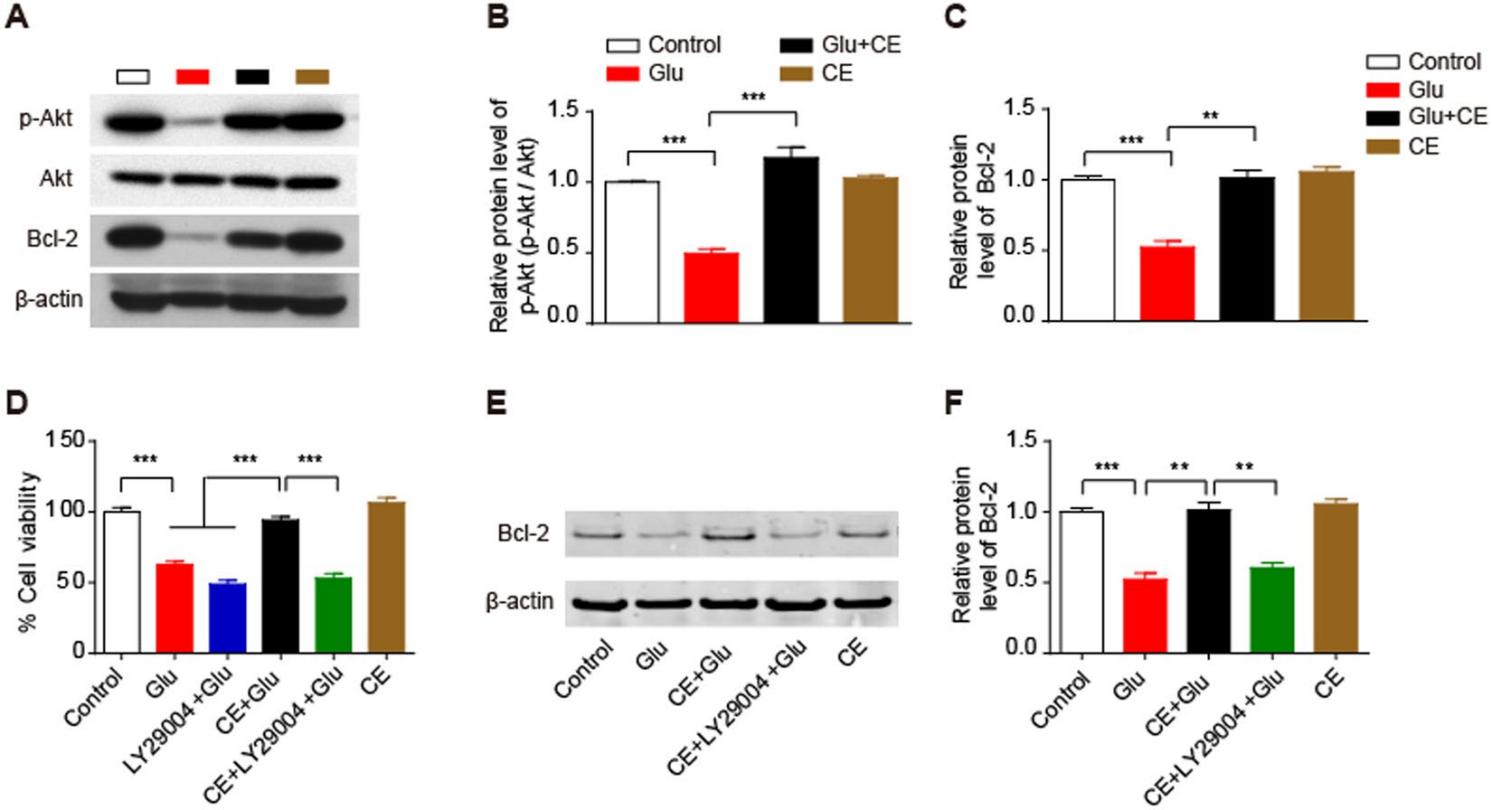
Involvement of the PI3K/Akt pathway in the neuroprotective effect of CE. (**A**,**B** and **C**) Cell lysates were analysed by immunoblotting to measure the relative levels of proteins involved in the Akt signalling pathway, including phosphorylated (Ser473) Akt and its downstream target Bcl-2 (**A**). Quantification of p-Akt and Bcl-2 protein levels is shown in (**B** and **C**), respectively. (**D**) Cell viability was measured using the MTT assay. In this experiment, cells were pretreated with the Akt inhibitor LY29004 for 30 min prior to treatment with CE or glutamate. (**E** and **F**) Immunoblot showing the Bcl-2 protein level (**E**); the intensity of the bands in each lane was quantified (**F**). (**B,C** and **F**), n = 3; D, n = 5; data are shown as mean ± SEM; One-way ANOVA followed by Tukey’s multiple comparisons test. **p < 0.01, ***p < 0.001.

The PI3K/Akt signalling pathway is associated with glutamate-induced excitotoxicity and is involved in neuroprotection^12–14^. We first detected the expression levels of phosphorylated Akt (S473) and its downstream target Bcl-2 to determine whether the PI3K/Akt pathway was involved in the neuroprotective effects of CE. As expected, the CE treatment remarkably inhibited the glutamate-induced decrease in the levels of phosphorylated Akt and its downstream target Bcl-2 (Fig. 4A–C). Moreover, the Akt inhibitor LY29004 completely blocked the CE-mediated protective effect on glutamate-induced excitotoxicity, as determined using the MTT assay (Fig. 4D), and CE did not rescue the glutamate-induced decrease in Bcl-2 expression in the presence of LY294002 (Fig. 4E and F). Thus, these results strongly support the hypothesis that the protective effect of CE on glutamate-induced excitotoxicity in cultured rat primary cortical neurons is medicated by Akt and its downstream target Bcl-2.

### CE regulates the PKC-GluA2 axis to exert its neuroprotective effects

**Figure 5:**
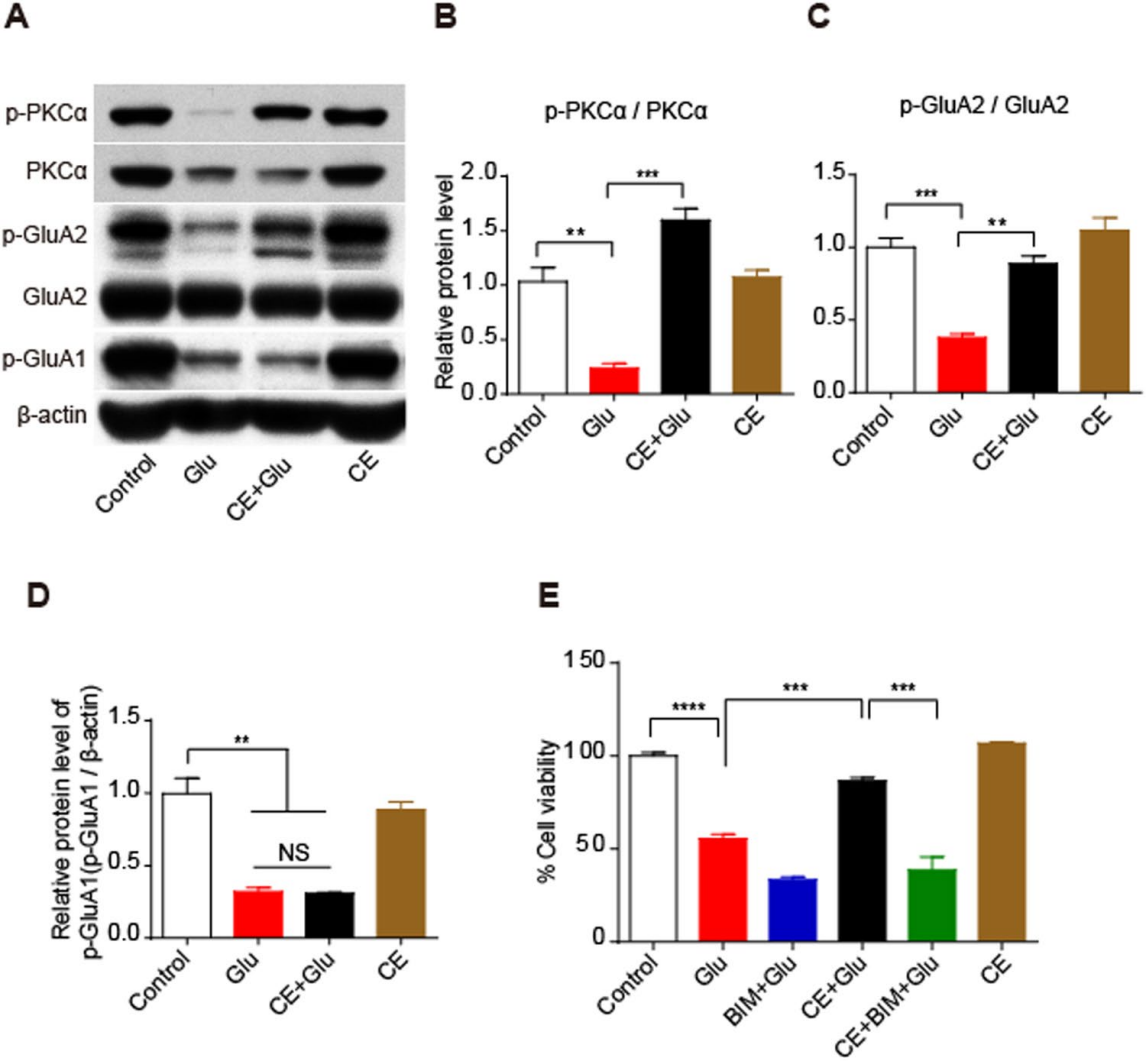
The PKC-GluA2 axis plays a key role in the neuroprotective effect of CE. (**A**) Immunoblotting showing the expression levels of the p-PKCα, PKCα, p-GluA2 and GluA2, and p-GluA1 proteins. Relative protein levels were quantified and shown in (**B**,**C** and **D**) respectively, n = 3. (**E**) Cell viability was measured using the MTT assay. Cells were pretreated with the PKC inhibitor Bisindolylmaleimide I (BIM) for 30 min prior to treatment with CE or glutamate, n = 5. Data are shown as mean ± SEM from 3 individual experiments; One-way ANOVA followed by Tukey’s multiple comparisons test. NS, not significant, **p < 0.01, ***p < 0.001.

Sustained stimulation of glutamate receptors (GluRs) by glutamate has been reported to lead to excitotoxicity^15^. Among the GluRs, AMPA receptors (AMPARs), which are composed of the subunits GluA1-GluA4, mediate fast excitatory synaptic transmission^16^. Functionally, GluA1, GluA3, and GluA4 display strong calcium permeability, whereas the GluA2 subunit reduces calcium permeability^17, 18^. In particular, PKC-mediated phosphorylation of GluA2 at S880 is associated with AMPAR endocytosis and LDP regulation^19, 20^. In this study, we investigated the potential involvement of AMPARs in CE-meditated neuroprotection against excitotoxicity. According to the western blot results, glutamate significantly decreased the phosphorylation of GluA1 (S845) and GluA2 (S880). Interestingly, the glutamate-induced dephosphorylation of GluA2 (S880) was almost completely rescued by the CE treatment, whereas CE did not exert effects on the phosphorylation of GluA1 (S845) (Fig. 5A,C and D). Moreover, we detected the expression level of PKCα (the protein kinase that phosphorylates GluA2 at S880). As shown in Fig. 5A and B, PKCα phosphorylation (p-PKCα, S657) was also significantly reduced following exposure to glutamate, and this effect was reversed by the CE treatment. In addition, a PKC-specific inhibitor, Bisindolylmaleimide I (BIM), was used to block the PKC-GluA2 pathway, and the CE-mediated neuroprotection was eliminated by the BIM treatment (Fig. 5E). Thus, CE may regulate the calcium impermeability function of the PKC-GluA2 axis to exert its neuroprotective effect.

### CE protects against neuronal loss in a mouse model of MPTP-induced PD

**Figure 6:**
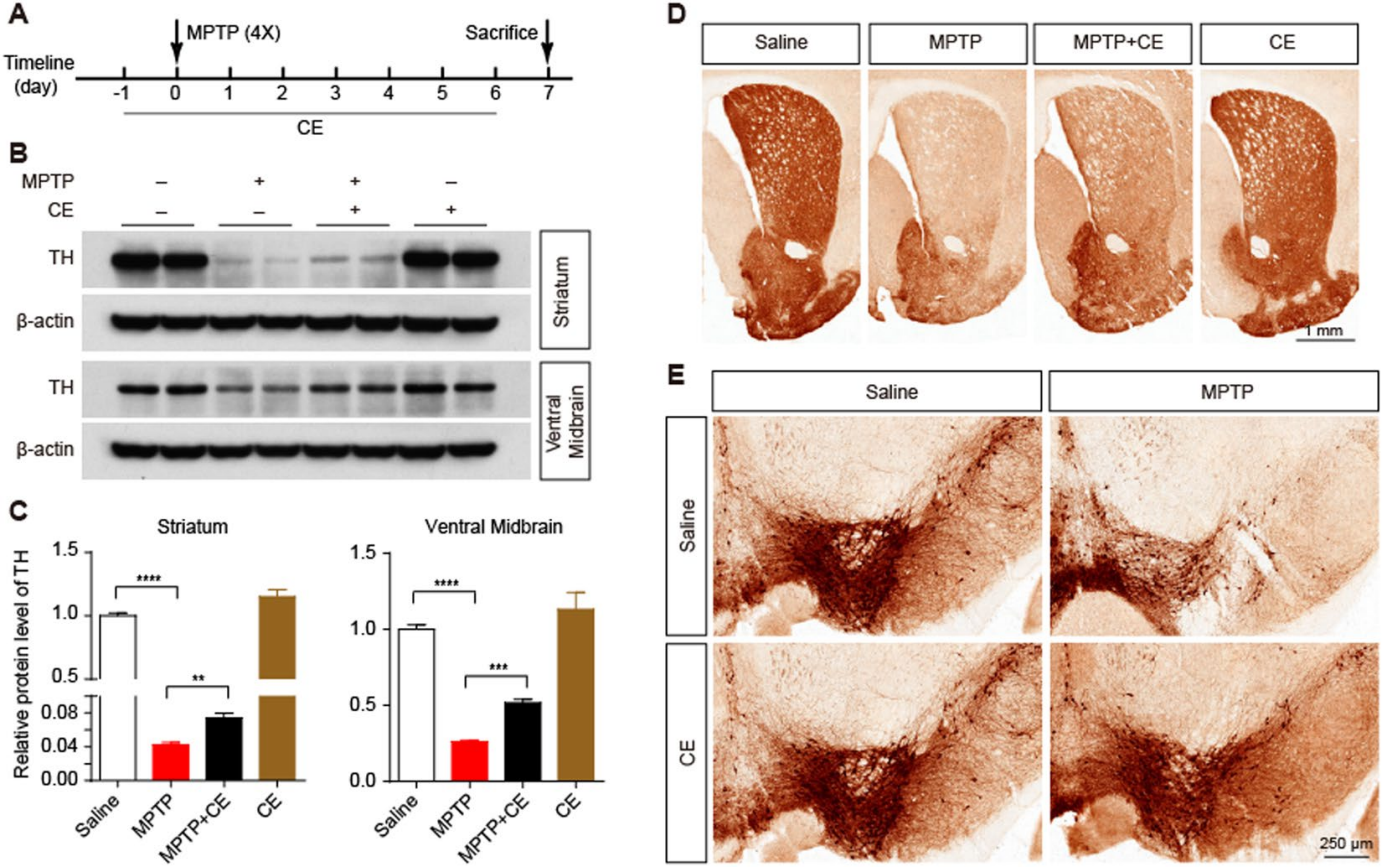
Neuroprotective effect of CE on a mouse model of MPTP-induced PD. (**A**) Schematic representation of the establishment of the PD mouse model and CE treatment. Adult C57BL/6 J mice were intragastrically administered 10 mg/kg CE or saline 12 h before receiving intraperitoneal injections of 20 mg/kg MPTP (4 times, 2 h intervals); mice were continuously treated with CE or saline until sacrifice. (**B**) Seven days after the MPTP injections, mice were sacrificed and the striatum and ventral midbrain tissues were dissected. The tissue lysates were immunoblotted with an anti-TH antibody to examine the loss of dopaminergic neurons. (**C**) Quantification of TH protein levels (normalized to β-actin). (**D** and **E**) The striatum and substantia nigra were visualized using immunohistochemical staining with an anti-TH antibody. Data are shown as mean ± SEM, n = 4 mice per group. One-way ANOVA followed by Tukey’s multiple comparisons test. **p < 0.01, ***p < 0.001. Scale bar, 1 mm (upper right) and 250 μm (bottom right).

Our previous studies^7, 8^ and current results have revealed a strong neuroprotective effect of CE against different insults (amyloid toxicity, oxidative stress and glutamate excitotoxicity) *in vitro*. A mouse model of MPTP-induced PD was used in this study to explore the *in vivo* effect of CE. As shown in Fig. 6A, mice were treated with CE or saline 12 h before MPTP injections (four intraperitoneal injections of 20 mg/kg at 2 h intervals) and continuously treated until sacrifice. Seven days after the MPTP injections, mouse brains were removed, the striatum and ventral midbrain were dissected for immunoblotting analysis and the brain sections were used for the immunochemistry assay. As expected, we observed a dramatic decrease in the TH protein levels in the striatum and ventral midbrain (Fig. 6B and C) in the MPTP-injected groups, indicating that the MPTP treatment induced dopaminergic neuronal loss. Interestingly, CE significantly rescued the loss of dopaminergic neurons induced by MPTP (Fig. 6B and C). These results were confirmed by the immunohistochemistry assay (Fig. 6D and E). Based on our results, CE protected against neuronal loss in a mouse model of MPTP-induced PD, suggesting that CE exerts a neuroprotective effect *in vivo*.

## Discussion

Neurodegenerative diseases are defined as hereditary and sporadic conditions that are characterized by progressive nervous system dysfunction. Although neurodegenerative diseases such as AD and PD have been extensively studied over the past several decades, effective treatments for these devastating diseases are not available, and the few available therapies only temporarily improve certain symptoms. One of the most important characteristics of many acute and chronic neurodegenerative diseases, which include stroke, brain trauma, AD, PD and HD, is neuronal cell death^21, 22^. Thus, the identification of new neuroprotectants that prevent neuronal death is a promising strategy to treat neurodegenerative diseases. In fact, natural products, such as curcumin and polyphenols, have recently been reported as new potential therapeutic targets for neurodegenerative diseases. Based on accumulating evidence, green tea polyphenols and curcumin exert neuroprotective effects against amyloid beta/glutamate/staurosporine-induced neurotoxicity *in vitro* ^9, 23, 24^. In the present study, the glutamate-induced death of cultured rat primary cortical neurons was significantly attenuated by a CE treatment, indicating that CE exerted a neuroprotective effect. The beneficial effects of CE were consistent with our previous findings that CE protects against amyloid toxicity and oxidative stress in cortical neurons^7, 8^. Moreover, CE also inhibits ischemia-induced neuronal cell death and cognitive impairment in rats^25^. Based on these findings, CE exerts beneficial effects against neuronal cell death.

In this study, we used a glutamate-induced neuronal cell death model to investigate the neuroprotective effect of CE and the underlying mechanism. The PI3K/Akt signalling pathway is involved in glutamate-induced excitotoxicity^12–14^. Accordingly, we examined the involvement of this pathway in the protective effects of CE against glutamate-induced excitotoxicity. As expected, CE rescued the glutamate-induced decrease in the levels of p-Akt and its downstream target Bcl-2, reduced the expression of the apoptosis-related protein cleaved-caspase3, and eventually promoted cell survival. In addition, the neuroprotective effect of CE was totally abolished by an Akt-specific inhibitor, LY29004. Thus, these results strongly support the hypothesis that the PI3K/Akt signalling pathway is involved in the neuroprotective effects of CE.

Glutamate is the major excitatory neurotransmitter in the nervous system and responsible for many physiological functions, including long term potential (LTP), long term depression (LTD), learning and memory^26^. However, the excessive release and accumulation of glutamate leads to neuronal dysfunction and neuronal loss, which is generally believed to be involved in the development of certain neurodegenerative diseases, including PD, AD and HD^2, 3^. In fact, increased glutamate signalling in the brain has been suggested to be an important mechanism underlying the dopaminergic neuronal death and in triggering the induction of programmed cell death in the animal model of PD^27^. Furthermore, glutamate antagonists have been shown to have anti-akinetic effects on animal models of Parkinson’s disease^28^. In the present study, the natural product CE partially rescued neuronal loss in an animal model of MPTP-induced PD and protected against glutamate excitotoxicity in primary cultured neurons, suggesting the potential usefulness of CE in regulating dopamine/glutamate dysfunction in PD.

Generally, glutamate-induced excitotoxicity is mainly attributed to the overstimulation of GluRs, particularly NMDARs and AMPARs, which results in excessive calcium influx in neurons and then triggers intracellular cascade reactions, including reactive oxygen species (ROS) production, lipid peroxidation and mitochondrial dysfunction, eventually leading to cell death^29, 30^. Among the GluRs, AMPARs mediate fast excitatory synaptic transmission^16^. Functionally, GluA1, GluA3, and GluA4 display strong calcium permeability, whereas GluA2 subunit reduces calcium permeability^17, 18^. In particular, PKA-mediated phosphorylation of GluA1 (S845) has been shown to promote GluA1 insertion at the cell surface and synaptic retention, increasing the probability of open channels and regulating LTP. Meanwhile, PKC-mediated phosphorylation of GluA2 (S880) is associated with AMPAR endocytosis and LDP regulation^19, 20^. In the present study, we investigated whether AMPARs were involved in the neuroprotective effects of CE against glutamate excitotoxicity. Glutamate decreased the levels of both p-GluA1 (S845) and p-GluA2 (S880) in cultured rat cortical neurons, which may be the main pathway involved in glutamate-induced neurotoxicity. Interestingly, the decrease in p-GluA2 (S880) levels was almost completely rescued by the CE treatment, whereas no significant change was observed in p-GluA1 (S845) levels. Thus, we speculated that CE might regulate the phosphorylation of GluA2, which blocks calcium overload in neurons and decreases glutamate-induced excitotoxicity. We further examined the upstream kinase of GluA2, PKCα, to confirm our hypothesis. We consistently observed that the decrease in the p-PKCα levels was reversed by the CE treatment, indicating that the PKC-GluA2 axis might play a crucial role in the neuroprotective effect of CE. This hypothesis was further supported by the data from experiments using the PKC-specific inhibitor BIM, which totally blocked the effect of CE. Based on our results, the PKC-GluA2 axis is involved in the neuroprotective effects of CE. However, a limitation of this study is that we did not clearly determine whether CE influenced glutamate excitotoxicity intracellularly or extracellularly. Therefore, studies investigating whether CE passes through the cellular membrane into the cytoplasm to exert its neuroprotective function would be interesting.

**Figure 7:**
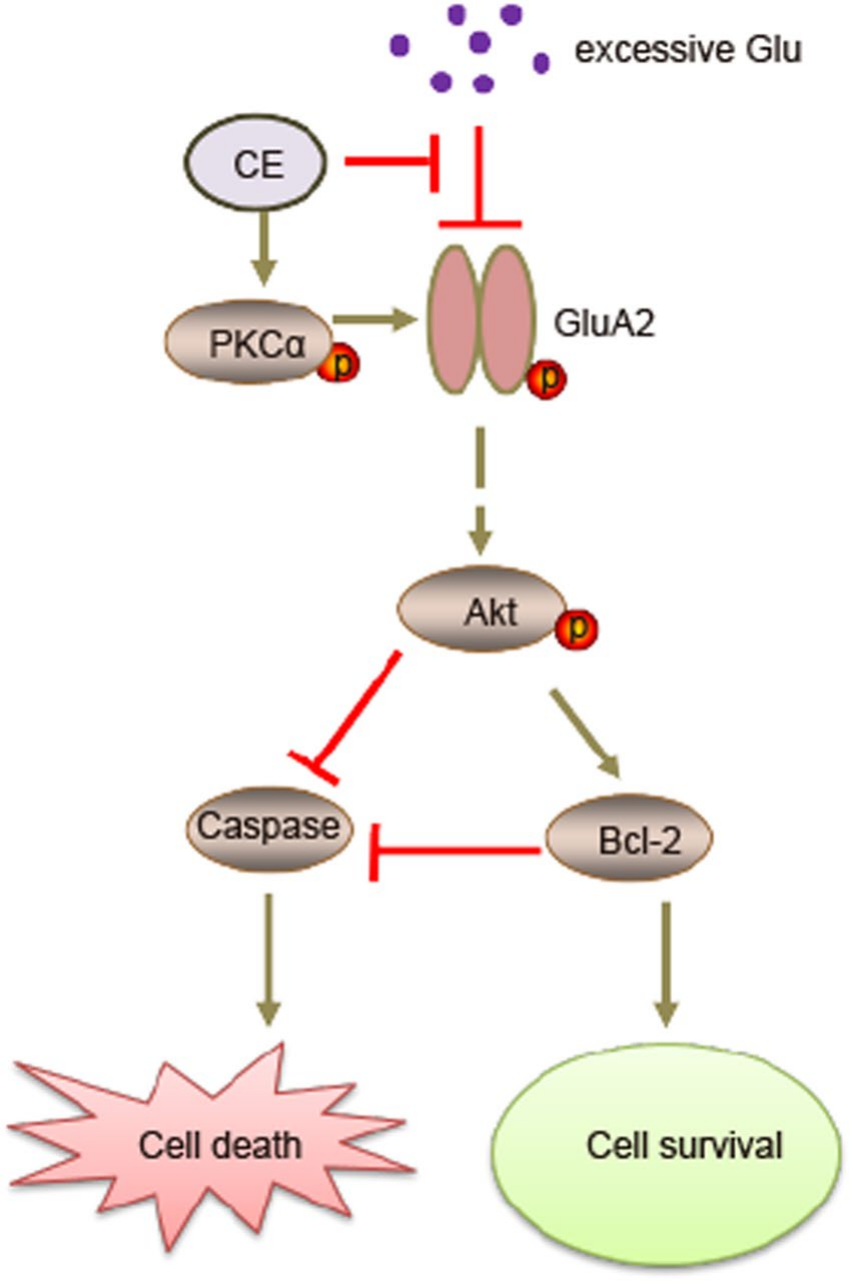
The proposed working model of the neuroprotective effect of CE. Excessive extracellular glutamate overstimulates GluRs and induces excitotoxicity, which is blocked by CE via the PKC-GluA2 axis. CE further regulates the Akt-Bcl-2-Caspase signalling pathway to prevent cell death and promote cell survival, exerting a neuroprotective effect.

In conclusion, CE prevented glutamate-induced excitotoxicity in cultured rat primary cortical neurons and the proposed model of CE-mediated neuroprotection is shown in Fig. 7. In addition, we also showed the neuroprotective effect of CE in a mouse model of MPTP-induced PD. Therefore, CE may represent a promising therapeutic strategy for the treatment of neurodegenerative diseases.

## Electronic supplementary material

Supplementary Information
