# Supplementary material for: Neuroprotective effects of a Coeloglossum viride var. Bracteatum extract in vitro and in vivo
Source: Sci Rep. 2017 Aug 23;7:9209. doi: 10.1038/s41598-017-08957-0 (PMC5569100; doi:10.1038/s41598-017-08957-0)

Supplementary Information

Neuroprotective effect of coeloglossum viride var. bracteatum extract *in vitro* and *in vivo*

Rui-Yuan Pan[1,2,3], Jun Ma[2,3], Huan-Tong Wu[1], Qing-Shan Liu[1] Xiao-Yan Qin[1*], Yong Cheng[1*]

[1] Center on translational neuroscience, College of Life & Environmental Science, Minzu University of China, Beijing 100081, China

[2] State Key Laboratory of Brain and Cognitive Sciences, Institute of Biophysics, Chinese Academy of Sciences, Beijing 100101, China

[3] College of Life Sciences, University of Chinese Academy of Sciences, Beijing 100049, China

Figure 2

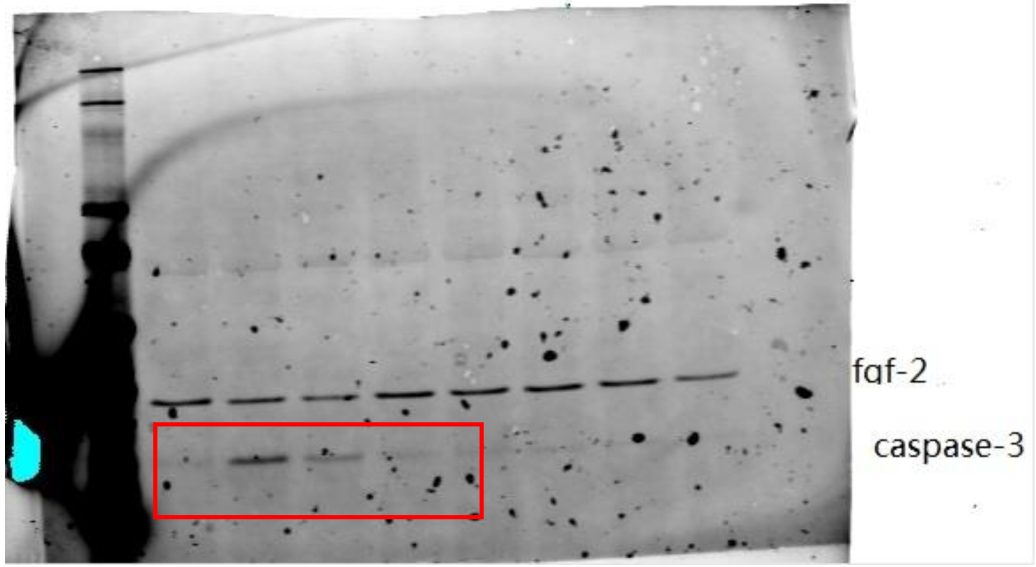

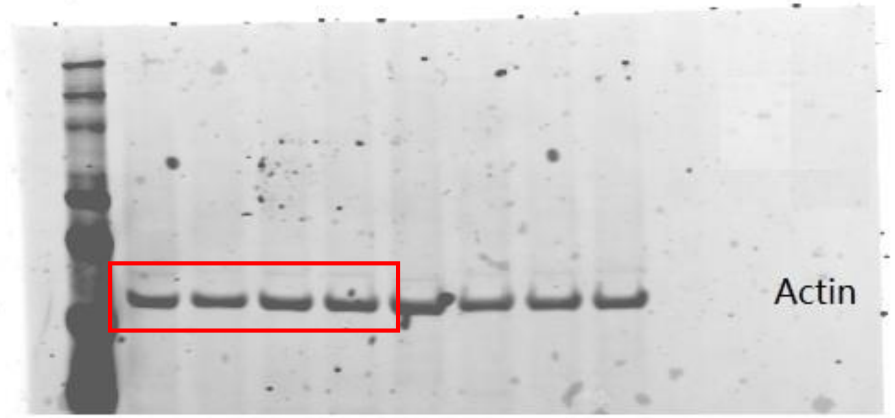

Figure 4

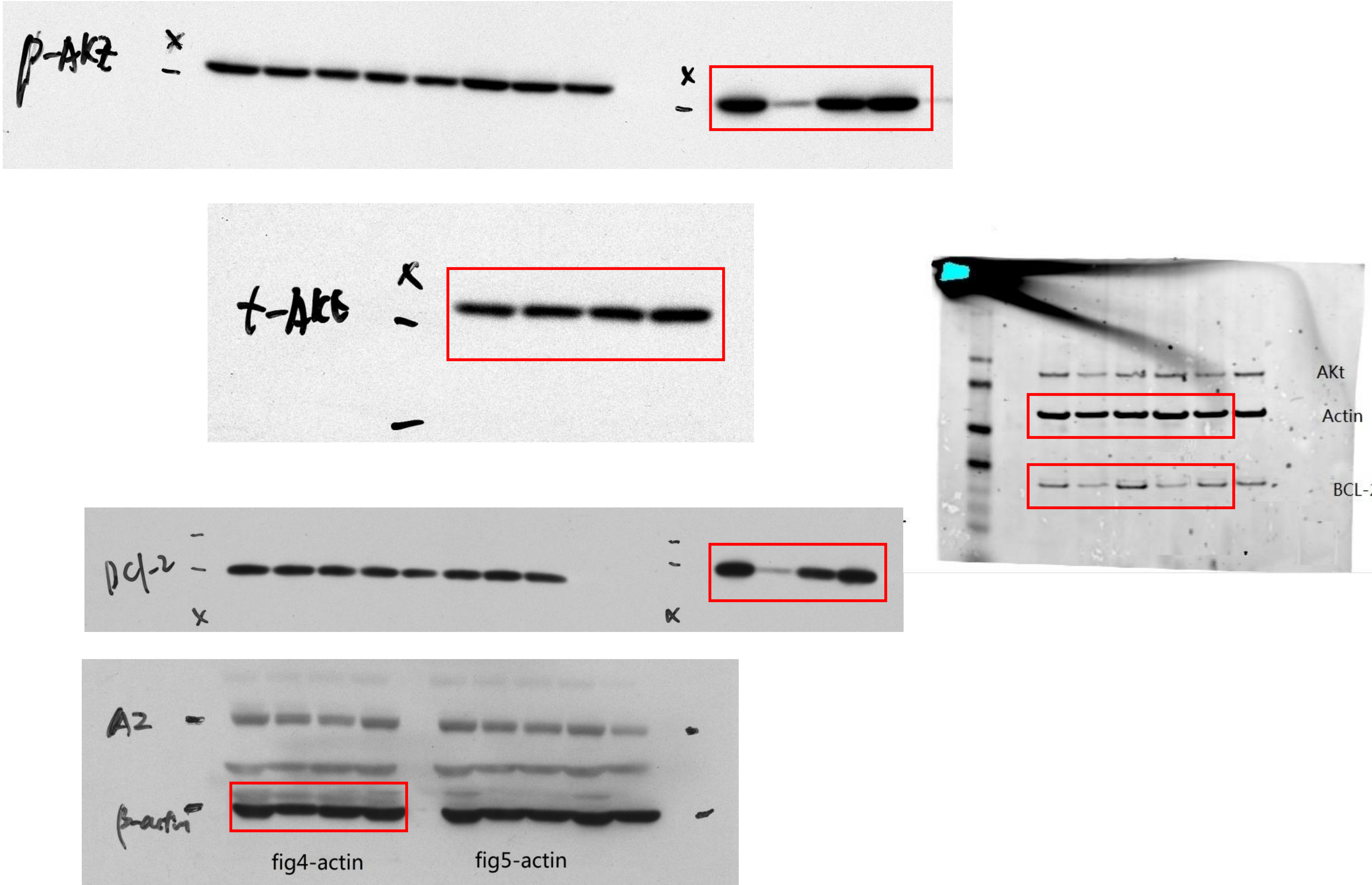

Figure 5

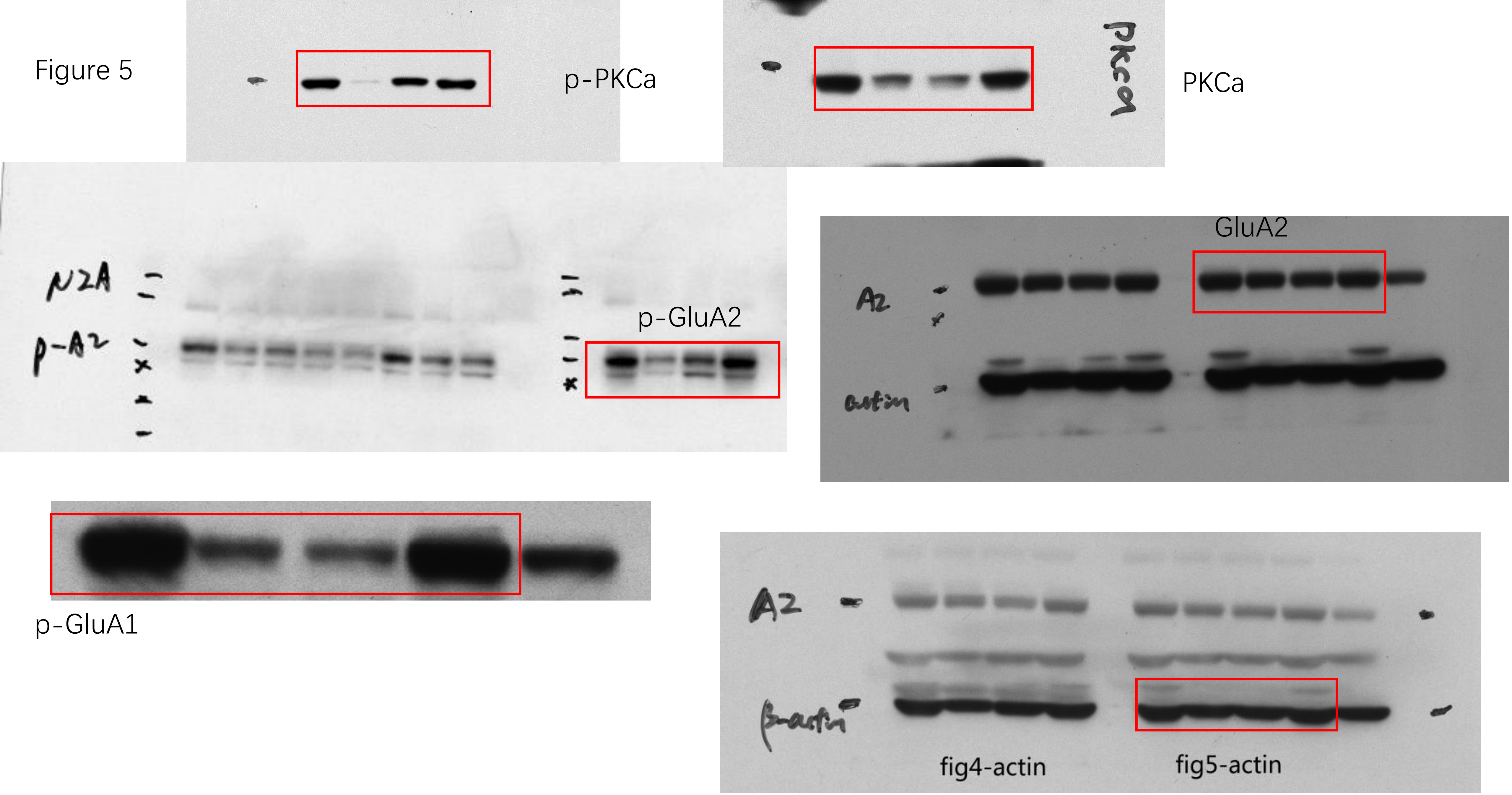
p-PKCa
PKCa
N2A
p-A2
p-GluA2
GluA2
A2
actin
p-GluA1
A2
β-actin
fig4-actin
fig5-actin

Figure 6

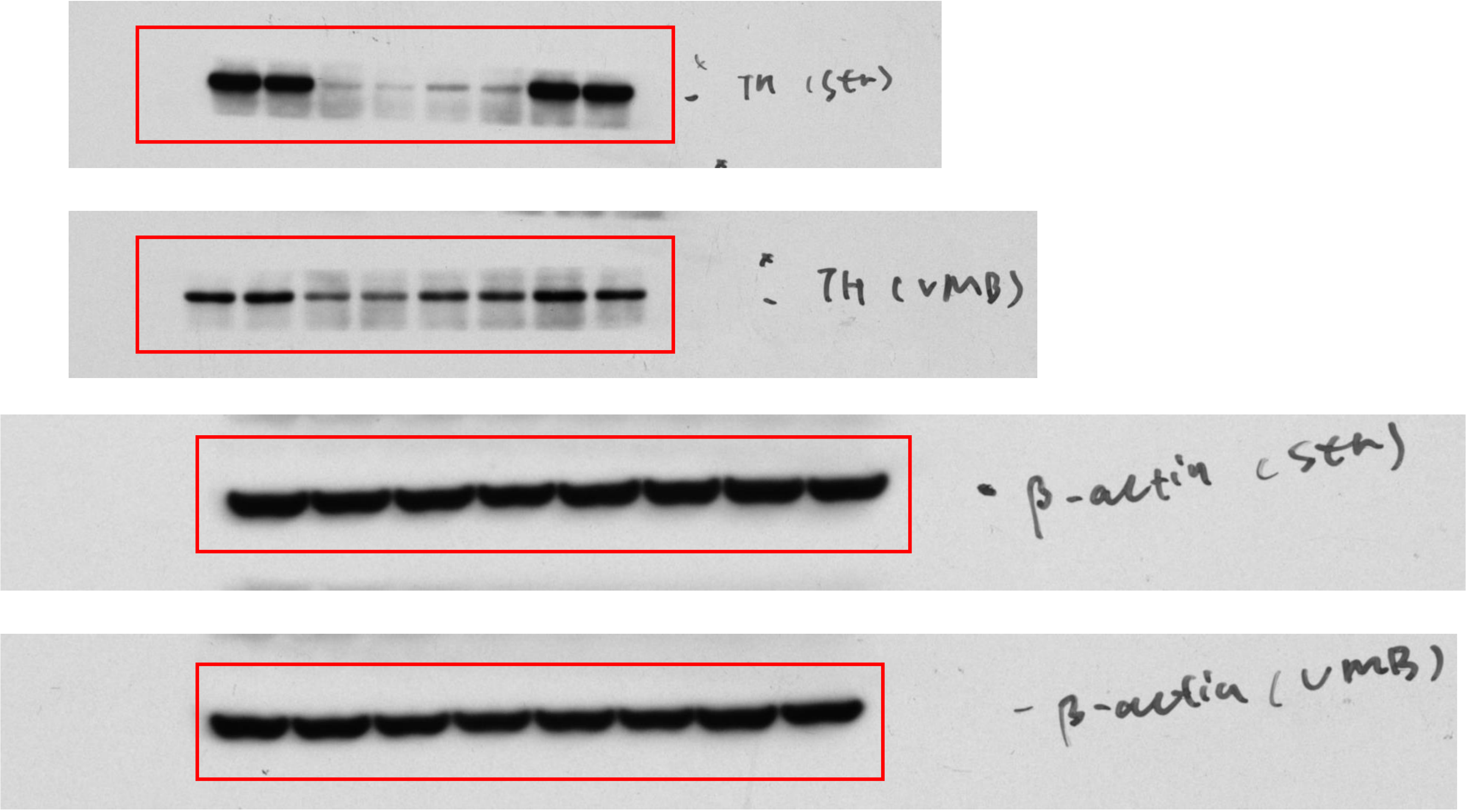

Supplement: Supplementary file 1 — Supplementary Information [file 41598_2017_8957_MOESM1_ESM.pdf]
